# Genetic polymorphisms of matrix metalloproteinases and their inhibitors in potentially malignant and malignant lesions of the head and neck

**DOI:** 10.1186/1423-0127-17-10

**Published:** 2010-02-15

**Authors:** Ajay Kumar Chaudhary, Mamta Singh, Alok C Bharti, Kamlesh Asotra, Shanthy Sundaram, Ravi Mehrotra

**Affiliations:** 1 Centre for Biotechnology, University of Allahabad, Allahabad, India; 2 Department of Pathology, MLN Medical College, Allahabad, India; 3 Division of Molecular Oncology, Institute of Cytology and Preventive Oncology (ICPO), NOIDA, India; 4 Tobacco-Related Disease Research Program, University of California Office of President Oakland, California, USA

## Abstract

Matrix metalloproteinases (MMPs) are a family of zinc-dependent proteinases that are capable of cleaving all extra cellular matrix (ECM) substrates. Degradation of matrix is a key event in progression, invasion and metastasis of potentially malignant and malignant lesions of the head and neck. It might have an important polymorphic association at the promoter regions of several MMPs such as MMP-1 (-1607 1G/2G), MMP-2 (-1306 C/T), MMP-3 (-1171 5A/6A), MMP-9 (-1562 C/T) and TIMP-2 (-418 G/C or C/C). Tissue inhibitors of metalloproteinases (TIMPs) are naturally occurring inhibitors of MMPs, which inhibit the activity of MMPs and control the breakdown of ECM. Currently, many MMP inhibitors (MMPIs) are under development for treating different malignancies. Useful markers associated with molecular aggressiveness might have a role in prognostication of malignancies and to better recognize patient groups that need more antagonistic treatment options. Furthermore, the introduction of novel prognostic markers may also promote exclusively new treatment possibilities, and there is an obvious need to identify markers that could be used as selection criteria for novel therapies. The objective of this review is to discuss the molecular functions and polymorphic association of MMPs and TIMPs and the possible therapeutic aspects of these proteinases in potentially malignant and malignant head and neck lesions. So far, no promising drug target therapy has been developed for MMPs in the lesions of this region. In conclusion, further research is required for the development of their potential diagnostic and therapeutic possibilities.

## Introduction

Carcinogenesis of the head and neck is a multi-step process. Head and neck malignancies consist of a heterogeneous group of neoplasia. They constitute the sixth most common malignancy, and more than 90% of these malignancies are squamous cell carcinoma (SCC) on histopathology. These are a significant cause of cancer worldwide. Incidence rates of these malignancies have been rising globally. It is estimated that 35,310 (25,310 males and 10,000 females) new cases of oral cavity and pharyngeal malignancies were diagnosed in the US during 2008, while 7,590 (5,210 males and 2,380 females) patients died of this disease [1]. The incidence of head and neck squamous cell carcinoma (HNSCC) has increased probably because of the increased use of tobacco and alcohol, which are widely documented as risk factors for this malignancy [2]. It has been reported that oral and oropharyngeal malignancies are the commonest carcinomas in males in North India and these account for about 30-40% of all cancer types in India - making it a leading cause of cancer mortality [3-5].

Tumour growth results from an imbalance between cell proliferation and apoptosis. It is influenced by angiogenesis, cell-cell and cell-extra cellular matrix (ECM) interactions. ECM consists of proteins and polysaccharides distributed in many different tissues of the body. ECM environment provides appropriate conditions for cell growth, cell differentiation and survival of tissues. It constitutes fibrous proteins such as collagen and elastin, elongated glycoproteins such as fibronectin and laminin, which provide cell matrix adhesion. The role of ECM in the tumour micro-environment is not limited to acting as a physical barrier to neoplasia, but it also works as a reservoir for ligand proteins and growth factors [6]. Matrix metalloproteinase are a family of zinc dependent endopeptidases that are capable of degrading most components of the extra cellular matrix (ECM) [7-9]. Degeneration of matrix is a key event in invasion and metastasis of malignant lesions of the head and neck.

Tissue inhibitors of matrix metallo-proteinases (TIMPs) are known to have the ability to inhibit the catalytic activity of MMPs. Gomez et al reported that in addition to the inhibitory role of TIMPs, they can also take part in the activation of MMPs [10]. TIMPs seem to have anti-angiogenic activity and they are also able to act as growth factors [11]. Turpeenniemi-Hujanen et al suggested that the expressions of matrix expression of MMPs as well as their tissue inhibitors the TIMPs are associated with the clinical behaviour in head and neck malignancy [12].

Many MMP promoter polymorphisms have been reported in malignant tissues such as in MMP-1 (-1607 1G/2G) [13,14], MMP-2 (-1306 C>T) [15] and MMP-7 (-181 A>G) [16] and these may be associated with susceptibility to invasive cervical carcinoma. McColgan et al recently, analyzed the polymorphic association of MMP-1 (-1607 1G/2G), MMP-2 (1306C>T, 735 C>T), MMP-3 and MMP-9 susceptibility to cancer in 30,000 subjects (with lung, breast and colorectal carcinoma). They reported no association with of MMP -1, -2, -3 or -9 polymorphisms with breast cancer, of MMP-1, -3 or -9 with lung cancer, or of MMP-2, -3 or -9 with colorectal cancer. Only MMP-1 (-1607 1G/2G) polymorphism was associated with colorectal cancer. The homozygous alleles for MMP-2 (-1306 or -735) polymorphism may, however, be responsible for a reduced risk of lung malignancy [17].

Li et al suggested that the G allele of the MMP-12 (82A/G) polymorphism might be a risk factor for the development and progression of epithelial ovarian carcinoma (EOC) and the A/A genotype of MMP-13 (-77A/G) polymorphism was associated with special pathological subtype and clinical stage in EOC in Chinese women population [18]. In an another study, Li et al genotyped MMP-12 -82G allele and MMP-13 -77A/G and suggested that these functional polymorphisms might play roles in developing gastric cardia adenocarcinoma (GCA) and esophageal squamous cell carcinoma (ESCC) in high incidence region of North China [19]. Recently, Peng et al suggested that MMP-1 (-1607 2G) may be associated with an increased cancer risk for colorectal carcinoma, HNSCC and renal carcinoma [20]. The present report aims to review the role, polymorphic association, gene expression of ECM and possible therapeutic aspects of MMPs and TIMPs in potentially malignant and malignant lesions of the head and neck.

### Classification of MMP gene family and their substrates

**Figure 1:**
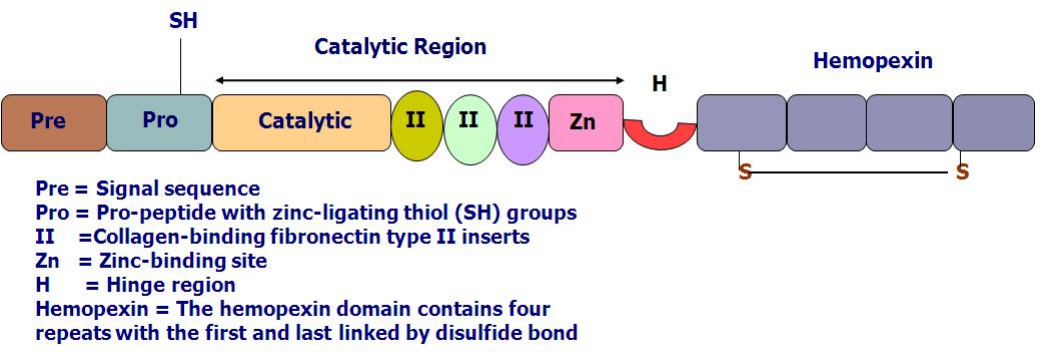
**Basic domain structure of the gelatinases (modified from Visse & Nagase 2003)**.

Currently, 24 different types of MMPs have been identified among vertebrates, 23 of them have been found in humans [21-23]. The members of the MMP family have many similarities in their structure. All MMPs have a zinc-binding motif in the catalytic domain. In addition, they have an N-terminal domain called predomain, followed by the propeptide domain. The majority of MMPs also have additional domains, e.g., Hemopexin domain. These additional domains are important in substrate recognition and in inhibitor binding (Fig 1).

MMPs can be divided into subgroups according to their structure and substrate specificity [21,23]. These subfamilies include collagenases, gelatinases, stromelysins, matrilysins, membrane-type MMPs (MT-MMPs) and other MMPs. Their substrates and chromosomal location are mentioned in Table 1. They are linked to ovulation, blastocyst implantation, embryonic development and tissue morphogenesis. They also play an important role in tissue repair, wound healing, nerve growth, mammary gland development, as well as, angiogenesis and apoptosis. All the proteolytic enzymes, potentially associated with tumour invasion, are members of the MMP family and are important due to their ability to degrade the ECM and basement membranes [24].

**Table 1:** Classification of vertebrate MMPs, their substrate and chromosomal location

Types of MMPs	Common Name	Chromosomal Location	Substrates
**MMP-1**	Collagenase-1	11q22.2-22.3	Collagen II<I<III,VII,VIII,X,XI,Casein, perlecan, entactin, laminin, pro-MMP-1,2,9,serpins
**MMP-8**	Collagenase-2	11q22.2-22.3	Collagen I>II>III>VII,VII,X,entactin,gelatin
**MMP-13**	Collagenase-3	11q22.2-22.3	Collagen II>III>I,VII,X,XVIII,gelatin,entactin,tenascin,aggregan
**MMP-18**	Collagenase-4	Not in humans	Collagen I,II,III,gelatin

**MMP-2**	Gelatinase-A	16q13	Gelatin, fibronectin, elastin, laminin, collagen I,III,IV,V,VII,X,XI
**MMP-9**	Gelatinase-B	20q11.2-q13.1	vitronectin,decorin,plasminogen Gelatin,CollagenI,IV,V,VII,X,XI,XVIII,vitronectin,Elastin,laminin,fibronectin, ProMMP-9 proMMP-2

**MMP-3**	Stromelysins-1	11q22.2-22.3	Laminin, aggregan gelatin, fibronectin
**MMP-10**	Stromelysins-2	11q22.2-22.3	CollagenI,III,IV,gelatin,elastin,proMMP-1,8,10
**MMP-11**	Stromelysins-3	22q11.2	Fibronectin,laminin,aggregan,gelatin

**MMP-12**	Metalloelastase	11q22.2-22.3	Elastin, gelatin, collagen I,IV, fibronectin, laminin, vitronectin, proteoglycan

**MMP-7**	Matrilysin-1	11q22.2-22.3	Collagen I,IV,V,IX,X,XI,XVIII, Fibronectin,laminin,gelatin,aggregan,,gelatin,proMMP-9
**MMP-26**	Matrilysin-2	11q22.2	Gelatin, Collagen IV,proMMP-9

**MMP-20**	Enamelysin	11q22	Laminin,amelogenin,aggregan

**MMP-14**	MT1-MMP	14q12.2	Collagen I,II,III,aggregan,laminin,gelatin,proMMP-2,13
**MMP-15**	MT2-MMP	16q12.2	Proteoglycans,proMMP-2
**MMP-16**	MT3-MMP	8q21	CoolagenIII,fironectin,proMMP-2
**MMP-17**	MT4-MMP	12q24	Gelatin,fibrinogen,proMMP-2
**MMP-24**	MT5-MMP	20q11.2	fibrinogen, Gelatin,proMMP-2
**MMP-25**	MT6-MMP	16q13.3	Collagen IV,gelatin,proMMP-2,9

**MMP-19**	Stromelysin-4	12q14	Collagen I,IV,Tenascin,Gelatin,Laminin
**MMP-21**	XMMP (Xenopus)	-	Gelatin
**MMP-22**	CMMP (Chicken)	-	-
**MMP23**	Cysteine array	1p36.3	Gelatin
**MMP-27**	(CA)	11q24	-
**MMP28**	CA-MMP Epilysin	17q11.2	Casein

(Modified from Sterlinct and Werb 2001; Overall 2002; Visse and Nagase 2003)

### Regulation of MMPs in potentially malignant and malignant head and neck lesions

The MMPs are regulated at many levels [21]. The expression of MMPs genes are transcriptionally induced by oncogenic transformation, cytokines as well as, growth factors - including interleukins, interferons, EGF, KGF, NGF, VEGF, PDGF, TNF-α and TGF-β [25]. The regulation of different MMPs also occurs at the protein level. MMPs are secreted as latent enzymes and this process can be achieved by activators and inhibitors. The expression of MMPs is primarily regulated at the level of transcription and their proteolytic activity requires zymogen activation. Many stimuli increase the expression of c-fos and c-jun proto oncogene products and it's activate the activator protein-1 (AP-1) at proximal promoter regions of several MMPs such as MMP-1, -3, -7, -9, -10, -12 and -13 types. Several oncogenes and viruses induce MMP expression in malignant cell lines [26]. MMP genes are induced by intracellular stimuli (MMP-1, MMP-3, MMP-7, MMP-9, MMP-10, MMP-12 and MMP-13) and bind an activator protein-1 (AP-1) at a binding site in the proximal promoter. In contrast, promoter region of MMP-2, MMP-11 and MMP-14 genes do not contain AP-1 elements [25]. Extra cellular signals activate the dimeric AP-1 complex which is composed of jun and fos proteins. These jun and fos proteins are bound to the AP-1 element and finally activate at the transcription level. Activity of AP-1 element is mediated by three groups of mitogen-activated protein kinases (MAPKs), which are mitogen-activated intracellular signal-regulated kinase 1, 2 (ERK1, 2), stress activated Jun N-terminal kinase and p38 MAPK [22]. The proteolytic activities of MMPs are inhibited by TIMP family (TIMP-1, -2, -3, -4) [27]. TIMPs inhibit the activity of MMPs by binding to activated MMPs. TIMPs can also inhibits the growth, invasion and metastasis of malignancies. Uzui et al reported that membrane type 3- matrix metalloproteinase (MT3-MMP) is expressed by smooth muscle cells (SMCs) and macrophages (Mphi) in human atherosclerotic plaques. Therefore, they suggested that the mechanism by which inflammatory molecules could promote Mphi macrophage-mediated degradation of ECM and thus therefore contribute to the plaque destabilization [28]. Thus, MMPs are regulated at the transcriptional and post-transcriptional levels and its control at the protein levels.

### Polymorphism of MMPs in potentially malignant and malignant head and neck lesions

A polymorphism is a genetic variant that appears in, at least, 1% of a population. Polymorphism represents natural sequence variants, which may occur in more than one form. Ra and Park suggested that approximately 90% of DNA polymorphisms are single nucleotide polymorphisms (SNPs) due to a single base exchange [29]. Common bi-allelic SNPs have been found in the promoter region of several MMPs. These promoter regions control transcription of gene function. Ye et al reported that the majority of polymorphisms are probably functionally neutral; a proportion of them it can exert allele (variant) specific effects on the regulation of gene expression. Such genetic polymorphisms are vital because they can be used as biomarkers that indicate for prognosis of potentially malignant and malignant lesions and thus may be involved in early intervention and diagnosis in patients at high risk. Levels of MMP gene expression can be influenced at the basal levels by genetic variations, susceptible to development or expression of several diseases [30].

#### MMP-1 promoter polymorphism

MMP-1 (Collagenase-1) is a major proteinase of the MMP family that specifically degrades type I collagen, which is a major component of the ECM, as well as other fibrillar collagens of types II, III, V and IX [31,32]. The MMP-1 gene is expressed in a wide variety of normal cells, such as stromal fibroblasts, macrophages, endothelial and epithelial cells, as well as, in various tumour cells [33]. Increased expression of MMP-1 has been associated with a poor prognosis in several malignancies such as colorectal carcinoma [34], bladder carcinoma [35], oral carcinoma [36,37] and nasopharyngeal carcinoma [38].

The MMP-1 gene is located on chromosome 11q^22 ^and the level of expression of this gene can be influenced by SNPs in the promoter region of their respective genes. Rutter et al suggested that a single nucleotide polymorphism at -1607 bp in the MMP-1 promoter contributes to increased transcription and cells expressing the 2 G polymorphism may provide a mechanism for more aggressive matrix degradation, thereby facilitating cancer progression [39]. The promoter region of MMP-1 contains a guanine insertion/deletion polymorphism (1G/2G polymorphism) at position -1607. Promoter assays have indicated that this is a functional polymorphism. Tower et al reported that this 2G allele results in increased transcriptional activity because the guanine insertion creates a core-binding site (5'-GGA-3') for the Ets transcription factor family, leading to a higher expression of MMP-1 [40].

Cao and Li genotyped 96 patients with oral squamous cell carcinoma (OSCC) and 120 controls, for 1G/2G polymorphism of MMP-1 (-1607) and reported that frequency of 2G allele was significantly higher in OSCC subjects (76%) than in the control group (56.7%) (OR = 2.2, 95% CI = 1.45-3.37, p = 0.00). They concluded that a SNP in the MMP-1 promoter -1607 was associated with OSCC susceptibility in the Chinese population [41]. Zinzindohoue et al investigated that impact difference of MMP-1 genotype in head and neck malignancies in a case control study (126/249) in a Caucasian population. Individuals homozygous for 2G/2G were at lower risk of developing malignancy than the 1G/1G carriers (OR = 0.37 95%CI = 0.19-0.71, p = 0.003). They concluded that haplotypic analysis showed a susceptibility of MMP-1 polymorphism in patients suffering from HNSCC [42]. Nishizawa et al examined the association of SNP in promoter regions of MMP-1 and MMP-3 with susceptibility to OSCC and found that frequency of MMP-1 2G alleles was higher as compared to 1G alleles (p = 0.03). Multivariate logistic regression analysis revealed that patients who were 45 years old or older had a 2.47 fold risk for development of OSCC (p = 0.0006) and suggested a crucial role of the MMP-1 2G allele in the early onset OSCC [37]. Vairaktaris et al suggested that MMP-1 -1607 1G/2G polymorphism increasing increased the risk for oral cancer in the 1G allele European carriers [43]. Hashimoto et al reported that the frequency of the MMP-1 2G/2G genotype was significantly higher in HNSCC patients than controls (140/223) (OR, 1.56, P = 0.042) and therefore concluded that the MMP-1 2G/2G genotype promoter polymorphism may be associated with HNSCC [44].

#### MMP-2 promoter polymorphism

MMP-2 was first identified and purified by Salo et al from metastatic murine tumours [45] and Höyhtyä et al cultured in human melanoma cells [46]. MMP-2 is a Zn^+2 ^dependent endopeptidase, synthesized and secreted in zymogen form. MMP-2 is tightly regulated at the transcriptional and post-transcriptional levels. Its primary function is degradation of proteins in the ECM. It is also able to degrade type IV collagen as well as type I, V, VII and X collagens, laminin, elastin, fibronectin and proteoglycans [47-49].

The MMP-2 gene is located on chromosome 16q^13 ^- (also known as Gelatinase-A). Functional SNP in the promoter region of MMP-2 has been reported and that may influence gene transcription and expression level in potentially malignant and malignant lesions. MMP-2 SNP is located at 1306 upstream of the transcriptional site and contains either a cytidine (C) or thymidine (T). Price et al reported that C>T transition at -1306, disrupts Sp1-binding site and results in decreased transcriptional activity, whereas the presence of the Sp1 promoter site in the -1306C allele may enhance transcription level [50]. Therefore, MMP-2 protein expression would be higher in individuals who carry the CC genotype than those who carry the TT or CT genotype.

O-Charoenrat and Khantapura examined the contribution of MMP-2 polymorphisms (-1306CT or TT) to susceptibility and aggressiveness of HNSCC. These polymorphisms, act as the promoters of MMP-2 (-1306 C>T) genotypes are capable of eliminating the Sp1-binding site and therefore down-regulate expression of the MMP-2 genes. They reported that subjects with the MMP-2 CC genotype was associated with significantly increased risk (OR, 1.97: 95% CI, 1.23-3.15) for developing HNSCC compared with those with the variant genotype (-1306 CT or TT). These findings suggested that the genetic polymorphisms in the promoters of MMP-2 may be associated with the development and aggressiveness of HNSCC [51]. Lin et al reported that MMP-2 -1306 C>T polymorphism in buccal squamous cell carcinoma (BSCC) and non buccal squamous cell carcinoma (NBSCC). CC genotype had nearly twofold increased risk for developing OSCC when comparing compared with CT or TT genotype, and CC genotype had more apparent risk (OR>4) for developing NBSCC [52] [Table 2].

**Table 2:** Functional Polymorphism of MMP-1(-16071G/2G), MMP-2 (-1306 C>T), MMP-3 (-1171 5A/6A) MMP-9 (P574R C>G;-1562 C>T) and TIMP-2 (-418 GC or CC) in potentially malignant and malignant head- and neck malignancies

Study	Country	Year	MMPs type	Mode of detection	Polymorphism	Case/Control group	OR	95%CI	p-value	Tumour
**Chaudhary et al **[57]	India	2010	MMP-3	PCR-RFLP	-1171 5A/6A	101/126 135/126	2.26 1.94	1.22-4.20 1.06-3.51	0.01 0.03	HNSCC OSMF

**Shimizu et al **[36]	Japan	2008	MMP-1 IL-8	PCR-RFLP, IHC	-1607 1G/2G IL-8-251 A/A	-	**-** **-**	**-** **-**	0.001 0.003	TSCC

**Wu et al **[61]	China	2008	MMP-9	PCR-RFLP	P574R C>G	-	4.1	1.58-10.52	0.00	ESCC

**Tu et al **[62]	Taiwan	2007	MMP-9	**PCR-RFLP**	-1562 C>T	192/191 73/191	-	**-**	0.029	OSCC OSMF

**Nasr et al **[38]	North Africa	2007	MMP-9 MMP-1	**PCR-RFLP**	-1562 C/T -1607 1G/2G	174/171	**-** 2.9	**-**	**-** 0.02	**-** NPC

**Vairaktaris et al **[63]	Greece	2008	MMP-9	**PCR-RFLP**	-1562 C/T	152/162	1.9	1.21-3.06	0.05	OSCC

**Vairaktaris et al **[55]	Greece	2007	MMP-3 MMP-1	**PCR-RFLP**	-1171 5A/6A -1607 1G/2G	160/156 141/156	2.2 -	1.0-4.5 -	< 0.05 < 0.05	OSCC

**Nishizawa et al **[37]	Japan	2007	MMP-1 MMP-3	**PCR-RFLP**	-1607 1G/2G -1171 5A/6A	170/164	2.4 -	1.5-4.6 -	0.000 -	OSCC

**O-Charoenrat and Khantapura **[51]	Thailand	2006	TIMP-2 MMP-2	**PCR-RFLP**	-418GC or CC -1306C-->T	239/250 239/250	1.43 1.97	0.98-2.08 1.23-3.15	- -	HNSCC

**Tu et al **[56]	Taiwan	2006	MMP-3	**PCR-RFLP**	-1171 5A/6A	150/98 71/98	1.7 2.62	0.84-3.445 1.20-5.71	0.18 0.01	OSCC OSF

**Cao and Li **[41]	China	2006	MMP-1	**PCR-RFLP**	-1607 1G/2G	96/120	2.2	1.5-3.4	0.000	OSCC

**Zinzindohoue et al **[42]	France	2004	MMP-1 MMP-3	**PCR-RFLP**	-1607 1G/2G -1171 5A/6A	126/249	0.37 -	0.2-0.7 -	0.003 -	HNSCC

**Lin et al **[52]	Taiwan	2004	MMP-2	PCR & dHPLC	-1306 C>T	121/147 58/147	2.0	**-**	**-**	OSCC OSF

**Hashimoto et al **[44]	Japan	2004	MMP-1 MMP-3	**PCR-RFLP**	-1607 1G/2G -1171 5A/6A	140/223	1.6 NS	- -	0.04 NS	HNSCC

[PCR-RFLP = Polymerase chain reaction-fragment length polymorphism, dHPLC = Denaturing high-performance liquid chromatography, ESCC = Esophageal squamous cell carcinoma, BSCC = Buccal squamous cell carcinoma, OSCC = Oral squamous cell carcinoma, OSMF = Oral sub-mucous fibrosis, NPC = Nasopharyngeal carcinoma, HNSCC = Head and neck squamous cell carcinoma, TC = Tongue squamous cell Carcinoma, NS = Not significant]

#### MMP-3 promoter polymorphism

The MMP-3 gene is located near the chromosome number 11q^22.2-22.3 ^and the level of expression of this gene can be influenced by SNPs in the promoter region of their respective genes. MMP-3 (stromelysin-1) lyses the collagen present in the basal membrane and induces synthesis of other MMPs such as MMP-1 and MMP-9 [33,53]. The promoter region of MMP-3 is characterized by a 5A/6A promoter polymorphism at position -1171 in which one allele has six adenosines (6A) and the second has five adenosines (5A). Ye et al reported that the 6A allele has a lower promoter activity than the 5A allele in vitro [30]. MMP-3 also plays a pivotal role in inflammation and thrombosis.

In addition, MMP-3 SNP has been reported to be associated with both susceptibility to and the invasiveness of breast cancer [54]. Increased levels of MMP-3 have been correlated with progression of oncogenesis and metastasis. Different findings have been reported by various workers. Vairaktaris et al investigated the possible association of -1171 5A/6A polymorphism, which influences high expression of 5A alleles of the MMP-3 gene in oral malignancy and reported a significant increase of 5A/6A heterozygote in OSCC patients as compared to control groups (p < 0.05). In addition, as a risk factor for smoking, the genotypes containing the 5A allele (5A/5A and 5A/6A) showed double risk of OSCC development. (OR = 2.16) [55]. On the other hand, Zinzindohoue et al reported that MMP-3 6A allele seemed to be associated with decreased risk of HNSCC [42]. Nishizawa et al reported that there was no difference in MMP-3 genotype distribution (5A/5A, 5A/6A, and 6A/6A) between the OSCC cases and control groups. (p = 0.188) [37] while, Tu et al concluded that the 5A genotype of MMP-3 promoter was associated with the risk of premalignant lesions like oral sub mucous fibrosis (OSMF) (P = 0.01) but not OSCC (P = 0.18) [56]. Recently, Chaudhary et al suggested that the expression of MMP-3 genotype associated with the 5A alleles may have an important role in the susceptibility to develop the OSMF and HNSCC in Indian population. We analyzed the MMP-3 (-1171 5A->6A) polymorphism; revealed the frequency of 5A allele in OSMF, HNSCC and controls group were 0.15, 0.13 and 0.07 respectively. In this study, 5A genotype had greater than two fold risk for developing OSMF (OR = 2.26) and nearly the same in case of HNSCC (OR = 1.94) as compared to controls. To the best of our knowledge, this is the first study dealing with MMP-3 polymorphism in OSMF and HNSCC patients of Indian origin [57].

#### MMP-9 promoter polymorphism

MMP-9 (gelatinase-B) was first synthesized by human macrophages [58] as well as pig polymorphonuclear leucocytes [59]. MMP-9 is a zinc-dependent endopeptidase, synthesized and secreted in monomeric form as zymogen. The structure is almost similar to MMP-2. MMP-9 gene proteolytically digests decorin, elastin, fibrillin, laminin, gelatin (denatured collagen) and types IV, V, XI and XVI collagen, as well as, activates growth factors like proTGFβ and proTNFα [60]. Elahi et al reviewed the genetics of the tumour necrosis factor-alpha (TNF-α-308) polymorphism in selected major diseases and evaluated its role in health and disease [61]. Physiologically there are only a few cell types expressing MMP-9 including trophoblasts, osteoclasts, leucocytes, dendritic cells and their precursors, and, in that respect, MMP-9 differs from MMP-2, which is expressed by a wide variety of cell types in normal conditions [21].

MMP-9 plays an important role in tumour invasion and metastasis by degrading ECM components. Variations in the DNA sequence in the MMP-9 gene may lead to alter its expression activity. The MMP-9 gene is located near the chromosome number 20q^11.2^-q^13.1^. Polymorphisms in the promoter of MMP-9 have been implicated in the regulation of gene expression and susceptibility to various diseases. The -1562 C>T polymorphism in MMP-9 promoter leads to differential transcription, and is associated with increased susceptibility to neoplastic and vascular diseases.

Wu et al investigated that the association of the MMP-9 polymorphisms and their haplotypes with the risk of esophageal SCC (ESCC) and significant differences were found in the genotype and allele distribution of P574R polymorphism of the MMP-9 gene as compared with the CC genotypes among cases and controls (OR = 4.08: 95% CI: 1.58-10.52: p = 0.00). They concluded that MMP-9 gene P574R polymorphism may contribute to a genetic risk factor for ESCC in the Chinese population [62]. Tu et al reported that no strong correlation of the MMP-9 expression is closely involved in tumour invasiveness and the prognosis of head and neck malignancies and that functional MMP-9 -1562 C>T polymorphism in the MMP-9 promoter with the risk of either is associated with OSCC or OSMF in male risk only in younger areca chewers [63]. Vairaktaris et al, from Greece, investigated MMP-9 -1562 C>T polymorphism and reported a strong association (OR = 92, 95%CI = 1.21-3.06, P < 0.05) with increased risk for developing oral cancer [64]. Nasar et al reported that no association in the genetic variations of MMP-9 polymorphism in nasopharyngeal carcinoma (NPC) [38].

Recently, Vairaktaris et al also examined the possible interactions between nine such polymorphisms, MMP-1 (-1607 1G/2G), MMP-3 (-1171 5A/6A), MMP-9 (-1562C/T), TIMP-2 (-418C/G), VEGF (+936C/T), GPI-α (+807C/T), PAI-1 (4G/5G), ACE (intron 16D/I) and TAFI (+325C/T) in an European population and concluded that four out of nine (PAI-1, MMP-9, TIMP-2 and ACE) polymorphisms affecting expression and contributed significantly leading factors to development of OSCC [65].

### Tissue inhibitors of metalloproteinase (TIMPs)

Tissue inhibitors of metalloproteinases (TIMPs) are naturally occurring inhibitors of MMPs which inhibit MMP activity and thereby restrict breakdown of ECM. By inhibiting MMP activity, they contribute to the tissue remodeling process of the ECM. The balance between MMPs and its tissue inhibitors plays a vital role in maintaining the integrity of healthy tissues. Disturbance in balance of MMPs and TIMPs is found in various pathologic conditions, including rheumatoid arthritis, periodontitis and cancer [66]. The role of TIMPs in potentially malignant and malignant lesions is very complex and ECM degradation is vital in spread of malignant cells and metastasis.

#### Expression of TIMP

Structurally, four different types of TIMPs have been characterized in man, designated TIMP-1, -2, -3 and -4. The genes that encode human TIMPs are mapped on X-chromosome number Xp^11.3 ^- Xp^11.23^, 17q^25^, 22q^12.1^-q^13.2 ^and 3p^25 ^respectively [67-69]. They show 30-40% similarity in structure at the amino acid level and possess 12 conserved cysteine residues required for the formation of six loops.

#### Polymorphism of TIMPs in potentially malignant and malignant head and neck lesions

O-Charoenrat and Khantapura examined the contribution of TIMP-2 polymorphisms (-418GC or CC) to susceptibility and aggressiveness of HNSCC. They reported that the TIMP-2 polymorphism showed a moderately increased risk of these malignancies and that was associated with the variant allele (-418GC or CC) compared with the GG common allele (OR, 1.43: 95% CI, 0.98-2.08). These findings suggested that the genetic polymorphisms in the promoters of TIMP-2 may be associated with the development and aggressiveness of HNSCC [51].

### Matrix metalloproteinase inhibitors (MMPIs) in cancer therapy

**Figure 2:**
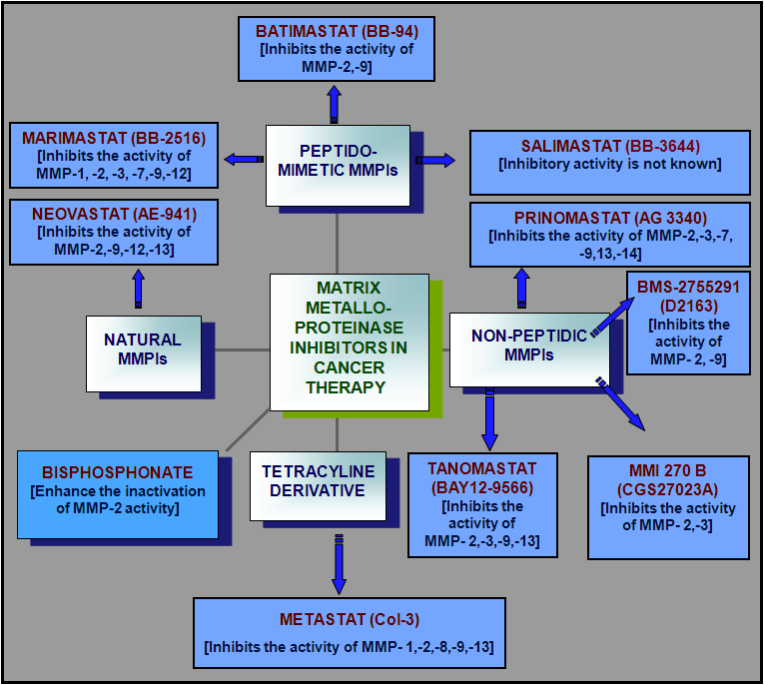
**Systematic representation of matrix metalloproteinase inhibitors (MMPIs) used in cancer therapy**.

Inhibition of MMP activity in the ECM has been involved in invasion of malignant cells. Shah et al reported functional degradation of ECM and therapeutic efforts to favorably alter the balance between MMP proteolysis and ECM synthesis [70]. Many MMPIs have been in clinical trials and are expected to present a new approach to cancer treatment. MMPIs may inhibit malignant growth by enhancing fibrosis around malignant lesions, by this means preventing tumour invasion, apoptosis and angiogenesis. Inhibitors of MMPs fall into five categories: (A) Peptidometric (B) Non-peptidometric (C) Natural MMPIs (D) Tetracycline derivatives and (E) Bisphosphonates [Fig. 2].

#### Peptidomimetic MMPIs

Three novel peptidomimetic phosphonate inhibitors have been synthesized and evaluated as potential inhibitors of MMP-2 and MMP-8. Peptidomimetic MMPIs are pseudo-peptide derivatives that mimic the structure of MMPs activity [49]. Hydroxyamate inhibitors are small (molecular weight < 6000) peptide analogs of fibrillar collagens, which inhibit MMP activity by specifically interacting with the Zn^2+ ^in their catalytic site. Most MMP inhibitors in clinical development are hydroxamate derivatives.

#### [A]Batimastat (BB-94)

Batimastat (BB-94) is a low molecular weight hydroxamate-based inhibitor that inhibits MMPs. It is a bioavailable low-molecular weight hydroxamate. It was the first MMPIs evaluated in cancer patients and to be used in a clinical trial. BB-94 inhibits the activity of MMP-2 and MMP-9. It is well tolerated, but its utility is limited because of its poor water solubility. Batimastat was administered by the intra-peritoneal and intra-pleural route for evaluation in clinical trials of the cancer patients [71-73]. Kruger et al reported that hydroxamate-type MMPIs BB-94 promotes liver metastasis in a mouse model [74]. Phase I and II clinical trials with intra-peritoneally administered BB-94 have not shown any marked response and at this time there is no further development of Batimastat (BB-94) for cancer therapy [75].

#### [B] Marimastat (BB-2516)

Marismastat (BB-2516) is a synthetic low molecular weight (331.4 D) peptidomimetic MMPI. Marismastat is an orally bioavailable and broad spectrum MMPI. It inhibits the genomic and proteomics activity of MMP-1, MMP -2, MMP -3, MMP -7, MMP -9 and MMP -12. The drug contains a collagen-mimicking hydroxamate structure that chelates the zinc ion at the active site of MMPs. Wojtowicz et al used Marismastat in a phase I clinical trial which was administered orally twice daily to 12 lung cancer patients and no consistent changes were seen in MMP level in blood [71]. Sparano et al concluded that patients on Marimastat do not have prolonged progression-free survival (PFS) when used after first-line chemotherapy for metastatic breast malignancy [76]. Tierney et al evaluated that safety and tolerability of 4 weeks of Marimastat administration in a phase I clinical trial in 35 patients with advanced gastro-oesophageal tumours, administering Marismastat once or twice daily for 28 days and found a favorable changes in these lesions [77].

Marimastat has been studied in phase II trials in patients with colorectal and advanced pancreatic cancer. It has been also studied in phase III clinical trials for treatment of pancreatic, ovarian, gastric and breast cancers as well as squamous cell lung carcinoma (SCLC) and non-squamous cell lung carcinoma (NSCLC). Overall survival of patients with advanced pancreatic cancer who were treated with Marimastat was not better than that of patients treated with Gemcitabine. Based on the outcome of these phase III trials results, evidence supported the use of MMPIs only in gastrointestinal malignancy. Zucker et al evaluated that the prognostic and predictive utility of measuring plasma levels of MMP-7 and MMP-9 in metastatic breast carcinoma (MBC) patients treated with the oral MMPI marimastat or a placebo and concluded that the plasma level of MMP-7 and MMP-9 was not a useful prognostic or predictive factor in patients with MBC or in patients treated with an MMPI [78].

#### [C] Salimastat (BB-3644)

Inhibitor activity of Salimastat (BB-3644) is not known. It has shown similar anticancer properties to Marimastat but failed in phase I clinical trial.

### Non-peptidic MMPIs

Non-peptidic MMP inhibitors have been sensibly synthesized on the basis of three dimensional X-ray crystallographic confirmation of MMP zinc-binding site. They are more specific and have better oral bioavailability than peptidometric inhibitors.

#### [A] Prinomastat (AG 3340)

AG 3340 is a synthetic, low molecular weight, nonpeptidic collagen-mimicking MMP inhibitor. It inhibits the activity of MMP-2, -3, -7, -9, -13 and -14. Hidalgo et al used this drug in a clinical trial in several xenograft models and concluded that Prinomastat inhibits tumour growth and angiogenesis [75].

#### [B] Tanomastat (BAY 12-9566)

Tanomastat (BAY 12-9566) is an orally bioavailable biphenyl compound. BAY 12-9566 and is a synthetic MMP inhibitor, which inhibits the activity of MMP-2, -3, -9 and MMP-13 [79]. It has been used in a phase III clinical trial in pancreatic, SCLC, NSCLC and ovarian cancer patients. The phase III clinical trials were cancelled because, in the SCLC trial, Tanomastat was performing less than placebo. On the basis of these findings, clinical progress of Tanomastat (BAY 2-9566) has also been suspended.

#### [C] BMS-2755291 (D2163)

BMS-2755291 is an orally bioavailable MMPI in phase I clinical development. It is an inhibitor of MMP-2 and MMP-9, which inhibits angiogenesis.

#### [D] MMI 270 B (CGS27023A)

MMI 270 B (CGS27023A) is wide range nonpeptidic inhibitors of MMPs. It is a strong inhibitor of MMP-1, MMP-2 and MMP-3. In a phase I clinical trial, 92 advanced solid cancer patients were treated and 20% of them reached stable disease. However, cutaneous rash and arthralgia were seen as side effects at high doses [80]. On the basis of these findings, clinical progress of CGS27023A has been suspended.

### Natural MMP Inhibitors

#### Neovastat (AE 941)

Neovastat is a natural MMP inhibitor and is orally bioavailable. It is extracted from shark cartilage. Function of Neovastat is based on multifunctional antiangiogenic effects. It inhibits the activity of MMP-2, MMP-9, MMP-12, MMP-13, elastase and function of vascular endothelial growth receptor-2 [81].

### Tetracycline derivatives

#### Metastat (col-3)

Metastat is a modified tetracycline derivative comprising a group of at least 10 analogues (CMT-1 to 10) on the basis of their MMP potency and specificity. It inhibits the activity of MMP-1, MMP-2, MMP-8, MMP-9 and MMP-13 and its down regulates the various inflammatory cytokines. Oral metastat is being evaluated in phase I clinical trials in cancer patients [82].

### Bisphosphonates

Bisphoshonates are a class of pharmacological substances and identified as MMP inhibitors. These are synthetic compounds with a high affinity for the hydroxyapatite crystal of bone. Their mechanism of action has not been completely confirmed. Their use in treatment of skeletal metastases in breast cancer and multiple myeloma has been established [83]. Farina et al concluded that Bisphoshonates prevent the inhibitory effect of TIMP-2 on MMP-2 degradation by plasmin and, by this means, enhance inactivation of MMP-2 activity [84].

## Conclusion

The molecular functions, expression, regulation and single nucleotide polymorphic association of MMPs such as MMP-1 (-1607 1G/2G), MMP-2 (-1306 C/T), MMP-3 (-1171 5A/6A), MMP-9 (-1562 C/T) and TIMP-2 (-418 G/C or C/C) and their role in head and neck malignancies have been reviewed. TIMPs are naturally occurring inhibitors of MMPs, which inhibit the activity of MMPs, therefore, control the breakdown of ECM. Useful markers associated with molecular aggressiveness might be of vital in predicting the conclusion of malignancies and to better recognize patient groups that need more aggressive treatment. Furthermore, the introduction of novel prognostic markers might promote exclusively new treatment possibilities and there is an undeniable need of markers that could be used as novel therapies as the existing therapies have made no difference in survival of these patients in the last 50 years. In conclusion, the MMPIs represent potential anticancer agents that are currently undergoing clinical trial in several potentially malignant and malignant diseases. There is no promising drug target therapy that has so far been evolved for the MMPs in potentially malignant and malignant lesions of the head and neck. Further research is required for the development of their potential diagnostic and therapeutic possibilities.

## Abbreviations

(MMP): Matrix metalloproteinase; (MMPI): Matrix metalloproteinase inhibitor; (SNP): Single nucleotide polymorphism; (PCR-RFLP): Polymerase chain reaction-restriction fragment length polymorphism; (OSMF): Oral submucous fibrosis; (HNSCC): head and neck squamous cell carcinoma.

## Competing interests

The authors declare that they have no competing interests.

## Authors' contributions

AKC carried out the analysis and prepared the manuscript. RM conceived of the study, participated in its design and coordination as well as helped to draft the manuscript. MS, SS, ACB and KA participated in coordination of the study and helped to draft the manuscript. All authors read and approved the final manuscript.
